# Camel Milk Mitigates Cyclosporine-Induced Renal Damage in Rats: Targeting p38/ERK/JNK MAPKs, NF-κB, and Matrix Metalloproteinases

**DOI:** 10.3390/biology10050442

**Published:** 2021-05-17

**Authors:** Hany H. Arab, Ahmed M. Ashour, Abdulmalik M. Alqarni, El-Shaimaa A. Arafa, Ahmed M. Kabel

**Affiliations:** 1 Department of Pharmacology and Toxicology, College of Pharmacy, Taif University, P.O. Box 11099, Taif 21944, Saudi Arabia; 2 Department of Pharmacology and Toxicology, College of Pharmacy, Umm Al Qura University, P.O. Box 13578, Makkah 21955, Saudi Arabia; amashour@uqu.edu.sa; 3 Department of Pharmaceutical Chemistry, College of Clinical Pharmacy, Imam Abdulrahman Bin Faisal University, Dammam 31441, Saudi Arabia; amalqarni@iau.edu.sa; 4 College of Pharmacy and Health Sciences, Ajman University, Ajman 346, United Arab Emirates; e.arafa@ajman.ac.ae; 5 Center of Medical and Bio-Allied Health Sciences Research, Ajman University, Ajman 346, United Arab Emirates; 6 Department of Pharmacology and Toxicology, Faculty of Pharmacy, Beni-Suef University, Beni-Suef 62514, Egypt; 7 Department of Pharmacology, Faculty of Medicine, Tanta University, Tanta 31527, Egypt; ahmed.kabal@med.tanta.edu.eg

**Keywords:** camel milk, cyclosporine, inflammation, MAPK, NF-κB

## Abstract

**Simple Summary:**

The widespread use of the immunosuppressant cyclosporine A in organ transplantation and autoimmune disorders has been associated with renal damage as an adverse effect. The present work aimed to examine the potential of camel milk, a natural agent with marked anti-inflammatory/antioxidant properties, to mitigate cyclosporine-induced renal injury. The present findings revealed that cyclosporine A induced severe renal damage in rats, as indicated by the increased levels of nephrotoxicity markers (serum creatinine, blood urea nitrogen, and kidney injury molecule 1). Moreover, cyclosporine A triggered a marked renal inflammation, as seen by increased renal pro-inflammatory cytokines (e.g., tumor necrosis factor-alpha, interleukin-1 beta, and interleukin-18), renal degradation enzymes (matrix metalloproteinases-2 and -9), and activation of renal inflammatory signaling pathways (mitogen-activated protein kinases and nuclear factor kappa B pathways). Furthermore, cyclosporine A instigated renal oxidative reactions and lowered the renal antioxidant defenses. Interestingly, camel milk (10 mL/kg/day; for 3 weeks by oral route) mitigated the aforementioned nephrotoxicity markers, pro-inflammatory cytokines, degradation enzymes, inflammatory pathways, oxidative stress, and augmented the renal antioxidant capacity. In conclusion, camel milk may be a promising natural agent that can inhibit cyclosporine-triggered renal damage.

**Abstract:**

Renal damage is a devastating adverse effect for cyclosporine; a widely used immunosuppressant drug. The present work examined the potential of camel milk, a natural agent with marked anti-inflammatory/antioxidant properties, to attenuate cyclosporine-induced renal injury. The kidney tissue was examined with the aid of Western blotting, immunohistochemistry, biochemical assays, including colorimetric and ELISA kits. The present findings revealed that camel milk (10 mL/kg/day; for 3 weeks by gavage) significantly lowered serum creatinine, BUN, and KIM-1 renal dysfunction markers. Mechanistically, camel milk inhibited renal inflammation, as seen by significant decrease of the pro-inflammatory cytokines (MCP-1, TNF-α, IL-1β, and IL-18) and extracellular degradation signals (MMP-2 and MMP-9) and enhanced the generation of the anti-inflammatory IL-10. Moreover, it inhibited the upstream pro-inflammatory p38/ERK/JNK MAPK pathway by lowering the phosphorylation of the 3 subfamilies of MAPKs (p38 MAPK, JNK1/2, and ERK1/2). Furthermore, camel milk curbed the NF-κB pathway activation by downregulating the protein expression of activated NF-κBp65, p-NF-κBp65, and p-IκBα proteins. Additionally, camel milk inhibited renal oxidative stress by lowering the MPO activity and augmenting the reduced/oxidized glutathione ratio and total antioxidant capacity. These findings propose that camel milk may be a promising agent that inhibits cyclosporine-triggered renal inflammation via curtailing the p38/ERK/JNK MAPK and NF-κB pathways, matrix metalloproteinases, and pro-inflammatory cytokines.

## 1. Introduction

The widespread use of the immunosuppressant cyclosporine A (CsA) in organ transplantation and autoimmune disorders, e.g., psoriasis and rheumatoid arthritis, has demonstrated marked clinical outcomes in patients with these ailments [1,2,3]. However, renal toxicity has emerged as a serious adverse effect, which progresses to chronic renal failure upon long-term administration [3]. In this context, the clinical data have revealed that CsA instigates a high incidence rate of renal damage upon administration in recipients with kidney/pancreatic transplant [4]. In non-kidney transplant patients, renal damage is regarded as the main trigger for renal failure and end-stage renal disorders [3,4]. 

The mechanisms for CsA-evoked nephrotoxicity are multifactorial, where inflammatory events play a dominant role in the pathogenesis of this disorder [3,5]. Classically, inflammation is regarded as an adaptive mechanism to rid cells of the invading insult and to allow time to rectify damaged tissues. However, the exaggerated inflammatory response and associated pro-inflammatory cytokine production, including interleukin 1 beta (IL-1β) and tumor necrosis factor-alpha (TNF-α), have been reported as pathogenic factors during the progression of renal damage. In perspective, upregulation of the inflammatory events has been characterized in CsA-induced nephrotoxicity [3,6]. Emerging evidence has demonstrated the involvement of mitogen-activated protein kinases (MAPKs) in cyclosporine insult [3,7]. In this context, p38 MAPK, c-Jun N-terminal kinase (JNK), and extracellular signal-regulated protein kinase (ERK) have been reported to mediate the CsA-induced glomerular dysfunction and renal damage [7,8]. Likewise, the nuclear factor-kappa B (NF-κB), a transcription factor for several pro-inflammatory signal genes, has been reported as a crucial target during the pathogenesis of CsA-evoked renal injury [5]. Notably, crosstalk has been described between MAPKs and NF-κB pathways, where activated ERK signaling results in the instigation of the NF-κB cascade in several renal pathologies, with consequent overshooting of pro-inflammatory cytokines [7,8]. Likewise, the activation of p38 MAPK has been established to perpetuate the inflammatory events via exaggerated cytokine generation. Of note, interventions that target multipronged pro-inflammatory cascades, such as MAPKs and NF-κB pathways, have been considered as effective tools for combating the CsA-induced renal damage [3,5]. 

Ample evidence exists that the long-term use of CsA has been associated with end-stage renal damage, and thus, finding novel agents that can intervene with the progression of CsA-evoked nephrotoxicity, without imposing adverse effects is highly demanded [3]. In this respect, camel milk has emerged as a natural agent with robust anti-inflammatory features, that were proven in in vitro and in vivo models. For example, the anti-inflammatory impact of camel milk mediated its favorable actions for the attenuation of inflammatory bowel disease [9], rheumatoid arthritis [10], steatohepatitis [11], and wound healing [12]. More important, the marked anti-inflammatory features of camel milk have also underlain its beneficial effects against several rodent models of renal injury, including diabetes mellitus- and chemotherapy-triggered nephropathy [13,14]. Notably, camel milk has demonstrated unique features beyond its nutritive impact, compared to cow milk. In this regard, camel milk is low in lactose, cholesterol, and β-lactoglobulin/β-casein [15]. Moreover, it possesses a unique insulin-like activity which saves 30–35% of insulin dose in patients with diabetes mellitus [16]. Furthermore, the rich content of lactoperoxidase and nanoantibodies has advocated its immune-modulatory, antibacterial, and antiviral features [15]. Compared to the milk of other species, including buffalo, cow, goat, sheep, and mare, camel milk is the richest in terms of the bioactive lactoferrin (220 mg/L), which imparts distinctive anti-inflammatory/antioxidant effects [17,18]. Despite the reported anti-inflammatory actions of camel milk in experimental models, its potential impact on the pathogenesis of CsA-evoked nephropathy and associated molecular mechanisms remains inadequately defined. Therefore, the current work aimed to investigate the potential anti-inflammatory actions of camel milk to limit CsA-induced nephrotoxicity and the molecular mechanisms pertaining to the inflammatory derangements, particularly, the p38/ERK/JNK MAPKs and NF-κB pathways and associated production of pro-inflammatory cytokines and matrix metalloproteinases.

## 2. Materials and Methods

### 2.1. Experimental Animals

The present work was conducted using male Wistar albino rats, 8-week-old, weighing 160–200 g. The animals were allowed unrestricted access to the lab food and drinking water, under constant experimental conditions of 12L/12D lighting system, 50–70% humidity, and 23–25 °C temperature. Before the start of the experimental protocol, animals were allowed a 10-day acclimatization span. 

The experimental protocol (Ethical Approval # 38-35-18, on 16 November 2016) was maintained under the procedures approved by the Taif University Research Ethical Committee, which also complied with the guidance of Laboratory Animal Guide for Care and Use established by US-NIH.

### 2.2. Chemicals

The camel milk (Al-Turath Al-Saudia Co., Jeddah, Saudi Arabia) was a commercial product with 3% (*w*/*v*) fat and 6.3% (*w*/*v*) non-fat solid content. The nephrotoxic agent cyclosporine was provided by a commercial vendor (Sigma-Aldrich Chemical Co., St. Louis, MO, USA).

### 2.3. Experimental Protocols 

Animal randomization was performed by a technician unaware of the experimental group identity. Five groups (each contain 8 rats) were employed to administer one of the following regimens daily, starting from the 1st day of the study till the 21st day, as follows: Group I (control group): Injection of the vehicle for cyclosporine (olive oil; s.c.; 1 mL/kg/day) plus oral vehicle (0.5% *w*/*v* carboxymethyl cellulose (CMC; 10 mL/kg/day) by gavage. Group II (control + CM 10): Injection of the vehicle for cyclosporine (olive oil; s.c.; 1 mL/kg/day) plus camel milk (10 mL/kg/day) by gavage. Group III (CsA group): Injection of cyclosporine (20 mg/kg/day; s.c.; 1 mL/kg/day) plus oral vehicle (0.5% CMC; 10 mL/kg/day) by gavage. Group IV (CsA + CM 10 group): Injection of cyclosporine (20 mg/kg/day; s.c.; 1 mL/kg/day) plus camel milk (10 mL/kg/day) by gavage. Group V (CsA + QRC group): Injection of cyclosporine (20 mg/kg/day; s.c.; 1 mL/kg/day) plus the reference antioxidant quercetin (50 mg/kg/day; 10 mL/kg/day) by gavage. Regarding the site for the subcutaneous injection, it was at the middle back region of rats, dorsal to the ribcage. The site of s.c. injection was changed throughout the study to minimize animal suffering.

The dose of cyclosporine and the experimental regimen was based on former studies that demonstrated the marked cyclosporine nephrotoxicity and robust renal inflammation [1,2,19,20]. Camel milk dose is consistent with former studies that demonstrated the 10 mL/kg dose as an effective dose against several models of inflammation in rats [10,13,14,15,16]. Likewise, the dose of quercetin has been reported as an effective dose against renal inflammation [21].

Support for the clinical relevance of the selected dose of camel milk for human subjects can be verified by calculating the human equivalent dose (HED) of camel milk, taking into consideration the relative body weight and the surface area of rats relative to adult human [22]. Following the conversion, the HED = animal dose × animal Km/human Km (the Km for the rat is 6 and the Km for human is 37), thus, the HED = 10 × 6/37 = 1.63 mL/kg for humans. Thus, for a 70 kg human, the HED is about 114 mL which is well-tolerated by human subjects.

### 2.4. Harvesting Serum and Renal Tissue

Following a treatment period of 21-days, rats were fasted overnight and anesthetized with sodium thiopental (50 mg/kg; i.p.). To harvest blood, a puncture to the orbital plexus of rat was performed with the aid of a capillary tube, and serum was separated. Then, rats were euthanized by cervical dislocation under anesthesia, and the kidneys were harvested and cleansed in saline. Serum and one kidney were kept at −80 °C for later biochemical analysis and immunoblotting. The other kidney (from 4 random animals per group) was preserved in 10% neutral-buffered formalin and was processed for paraffin embedding for later analysis by immunohistochemistry. 

For performing the ELISA determinations, part of the kidney was homogenized in RIPA buffer complemented with protease inhibitors cocktail to give a 10% *w*/*v* homogenate. The homogenate was maintained at 4 °C for 15 min, then centrifuged at 15,000× *g* at 4 °C and the supernatant was stored at −80 °C till performing the assays. Likewise, a 10% *w*/*v* homogenate in ice-cold saline was prepared and the homogenate supernatant was utilized for performing the TAC and GSH/GSSG ratio assays. 

### 2.5. The Nephrotoxicity Markers (Serum Creatinine and BUN and Renal KIM-1)

To examine the nephrotoxicity markers in serum, the levels of creatinine and blood urea nitrogen (BUN) were assayed using commercial vendor kits (Stanbio, Boerne, TX, USA). Moreover, the renal content of the kidney injury molecule-1 (KIM-1) was measured using Cusabio ELISA kit (Cusabio Biotech, Wuhan, China), according to the vendor instructions. The developed color for the assay was read at 450 nm. For performing each parameter, 8 samples were used per group. For each sample, the assay was carried out in duplicate. 

### 2.6. Measurement of Renal Cytokines

Specific ELISA kits were used for determining the renal content of interleukin-10 (IL-10), interleukin-1β (IL-1β), and tumor necrosis factor-α (TNF-α; RayBiotech, Peachtree Corners, GA, USA), interleukin-18 (IL-18; LifeSpan Biosciences, Seattle, WA, USA), and monocyte-chemoattractant protein-1 (MCP-1; Cusabio Biotech, Wuhan, China). The assay protocols were carried out as instructed by the provider. For performing each parameter, 8 samples were used per group. For each sample, the assay was carried out in duplicate.

### 2.7. Western Blotting of p-NF-κBp65, p-IκBα, p-p38 MAPK, p-ERK1/2, and p-JNK1/2

The renal tissue was homogenized/lysed in RIPA buffer supplemented with phosphatase/proteinase inhibitor cocktail [23]. For the immunoblotting, 30 µg of lysate protein was loaded and subjected to SDS-PAGE. The resolved proteins were blotted to PVDF membranes. The blots were cut before membrane blocking and probing with the target antibody against the phosphorylated protein, total protein, or β-actin. The membranes were blocked using 5% BSA, then, incubation of membranes with the specific primary antibody was performed at 4 °C overnight: anti-phospho-nuclear factor-kappa B protein 65 (p-NF-κB p65 (Ser536); 1:1000), anti-phospho-inhibitor of nuclear factor-kappa B alpha (p-IκBα (Ser32); 1:1000), anti-phospho-Jun N-terminal kinase 1/2 (p-JNK1/2 (Thr 183/185); 1:1000), anti-total JNK1/2 (1:2000), anti-phospho-p38 mitogen-activated protein kinase (p-p38 AMPK (Thr180/Tyr182); 1:5000), anti-total p38 MAPK (1:10,000), anti-phospho-extracellular signal-regulated kinase 1/2 (p-ERK1/2 (Thr202/Tyr204); 1:4000), and rabbit anti-total ERK1/2 (p44/42; 1:10,000, Cell Signaling Technology, Danvers, MA, USA). The blots were incubated with the appropriate secondary antibody (1:2000) for 1 h at ambient room temperature the next morning. Equal loading/protein transfer was proven by probing with anti-β-actin (1:5000, Cell Signaling Technology, Danvers, MA, USA). The target protein bands were visualized with BioRad Clarity Western ECL (BioRad, Hercules, CA, USA) and exposure to X-ray film (Hyperfilm-ECL, Amersham/GE Healthcare, Chalfont, UK). The X-ray films were photographed with an HD-Nikon camera and the densitometric analysis of protein bands was carried out using Image J software (Bethesda, Rockville, MD, USA) [24]. The Western blotting data were generated from 3-independent samples per group. The detection of the phosphorylated and total form of proteins was performed using separate gels. The detection of the abundant housekeeping β-actin protein was performed by stripping the primary/secondary antibodies specific for the phosphorylated or total proteins (using Restore Stripping Buffer, ThermoFisher, Cambridge, MA, USA) and reprobing the membranes using anti-β-actin primary antibody, followed by secondary antibody.

### 2.8. Immunohistochemical Staining of NF-κBp65, MMP-2, and MMP-9

The kidney sections (4-µm) on glass slides were blocked with 5% BSA [25]. In a moist chamber, the target proteins were stained using specific primary antibodies at 4 °C overnight: using anti-nuclear factor-kappa B protein 65 (NF-κBp65; 1:200 dilution). The degradation metalloproteinase signals were probed with anti-matrix metalloproteinase 2 (MMP-2; 1:100 dilution), and anti-matrix metalloproteinase 9 (MMP-9; 1:100 dilution; ThermoFisher Scientific, Cambridge, MA, USA). Then, the sections were subjected to HRP-labelled secondary antibody for 2 h and the protein antigens were visualized using DAB (Sigma-Aldrich, St. Louis, MO, USA) with counterstaining of sections using hematoxylin. The sections were examined under a light microscope complemented with a full-HD microscopic imaging system (Leica Microsystems, Wetzlar, GmbH, Germany). The digital pictures were analyzed for positive protein staining using Image J software (Bethesda, MD, USA) to quantify the target protein expression [26]. Quantification of target protein was performed by detecting the optical density of the brown immunostaining. The data of the immunohistochemistry were extracted from 4 samples from each group. The immunostaining was examined across 6 non-overlapping fields per section (×400 magnification).

### 2.9. Measurement of Renal Oxidative Stress Markers (MPO, GSH/GSSG Ratio, and TAC)

A kinetic assay was performed for determining the renal activity of myeloperoxidase (MPO) enzyme, as established by Krawisz et al. [27]. The O.D. of the final product was kinetically monitored at 460 nm. A colorimetric kit was used for the measurement of renal total antioxidant capacity that was procured from a commercial vendor (TAC; Cayman, Ann Arbor, MI, USA), as instructed by the provider. The TAC represents the whole set of tissue antioxidant moieties which guard against the attack triggered by reactive oxygen species (ROS). Thus, TAC may offer an index for the cumulative impact of the kidney antioxidants that combat oxidative stress. In this regard, the TAC assay depends on the principle that all the antioxidants in the kidney homogenate supernatant block metmyoglobin-triggered oxidation of azino-di(ethylbenzathiazoline sulfonate; ABTS). This was carried out by measuring the ability of total antioxidants in the sample to elicit a decline of the absorbance of the colored chromogen at 405 nm which is directly proportional to the level of total antioxidants in the sample. This ability was compared to the water-soluble tocopherol analog Trolox and the results were expressed as µmol Trolox equivalent per g tissue. 

The assay of reduced glutathione/oxidized glutathione (GSH/GSSG) ratio is an index for glutathione status in kidney homogenate. Briefly, Sigma-Aldrich colorimetric kit was used, as instructed by the provider [28]. First, the total glutathione level was assayed in the homogenate supernatant using glutathione reductase and dithiobis-nitrobenzoic acid (DTNB) reagent at 412 nm. Then, the oxidized form of glutathione was measured at 412 nm using DTNB reagent following the addition of a masking agent for the reduced form of glutathione. The reduced form of glutathione was calculated by the formula = total glutathione − oxidized form of glutathione. For performing each parameter, 8 samples were used per group. For each sample, the assay was carried out in duplicate. 

### 2.10. Analysis of Data and Statistics

The statistical analysis was carried out using GraphPad Prism software (version 6.00; San Diego, CA, USA). The data were checked to follow a normal distribution with the aid of Shapiro–Wilk normality test. Values were presented as mean ± SEM and the multiple comparisons across experimental groups were checked with the aid of one-way ANOVA, followed by Tukey–Kramer post-hoc test. For the analysis of data, the probability values (*p* < 0.05) were considered minimally accepted as significant. 

## 3. Results

### 3.1. Camel Milk Improves Renal Dysfunction Triggered by CsA in Rats

At the end of the experimental period, cyclosporine (20 mg/kg/day, s.c.) administration for 3 weeks induced a significant loss of body weight, relative to the control group (Table 1). The administration of camel milk (10 mL/kg/day; p.o., for 3 weeks) significantly inhibited the loss of body weight, relative to the renal injury group. This action was similar to the effect elicited by the reference quercetin (50 mg/kg/day, p.o., for 3 weeks). To explore the renal function alterations triggered by CsA, the levels of serum creatinine and blood urea nitrogen (BUN), along with the renal protein expression of kidney injury molecule-1 (KIM-1) was assayed. Relative to the control group, CsA administration instigated a marked renal dysfunction, as proven by significant elevation of serum creatinine (260%), BUN (280%), and KIM-1 renal protein expression (260%; Table 1), which is regarded as a sensitive nephrotoxicity marker [29]. When animals were treated with camel milk, the alterations of the kidney function markers were counteracted and brought back to near normal values. Interestingly, the lowering of the kidney function markers elicited by camel milk was similar to quercetin. Of note, camel milk administration alone to the control rats did not display significant alterations of the mentioned kidney function tests. Concerning the survival of rats in the present study, all animals survived the whole experimental period and no mortality was observed in the studied groups. Together, these findings confirm the efficacy of camel milk to improve the renal dysfunction evoked by CsA. 

### 3.2. Camel Milk Curbs the Pro-Inflammatory Responses Triggered by CsA in Rats 

**Figure 1:**
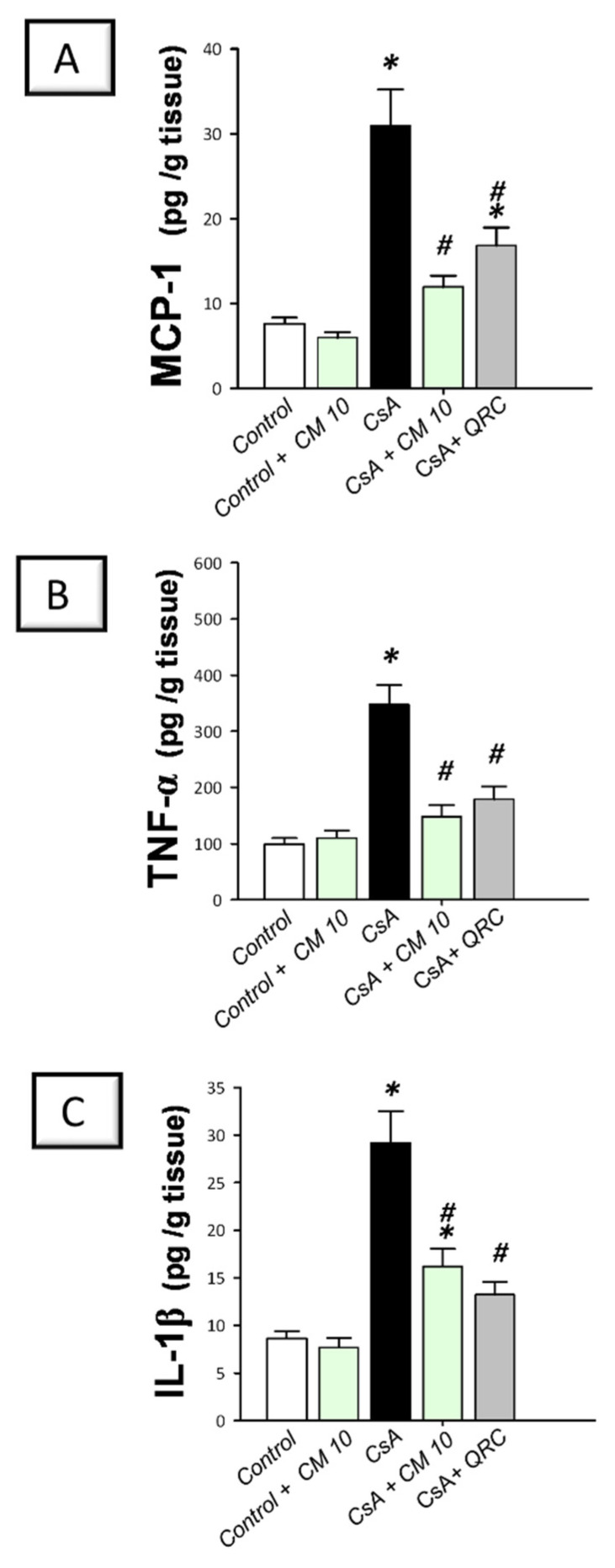
Effect of camel milk administration on renal MCP-1, TNF-α, and IL-1β pro-inflammatory cytokine levels in cyclosporine-evoked renal damage in rats. (**A**) Camel milk lowers the renal level of monocyte chemoattractant protein-1 (MCP-1). (**B**) Camel milk lowers the renal level of tumor necrosis factor-α (TNF-α). (**C**) Camel milk lowers the renal level of interleukin-1β (IL-1β). Values are displayed as mean ± SEM, for *n* = 8 samples per each group (one sample from each rat). * Significance vs. control values at *p* < 0.05; *^#^* Significance vs. CsA values at *p* < 0.05. CsA, cyclosporine (20 mg/kg/day, s.c., for 3 weeks); CM 10, camel milk (10 mL/kg/day, by gavage, for 3 weeks); QRC; the reference antioxidant quercetin (50 mg/kg/day, by gavage, for 3 weeks).

**Figure 2:**
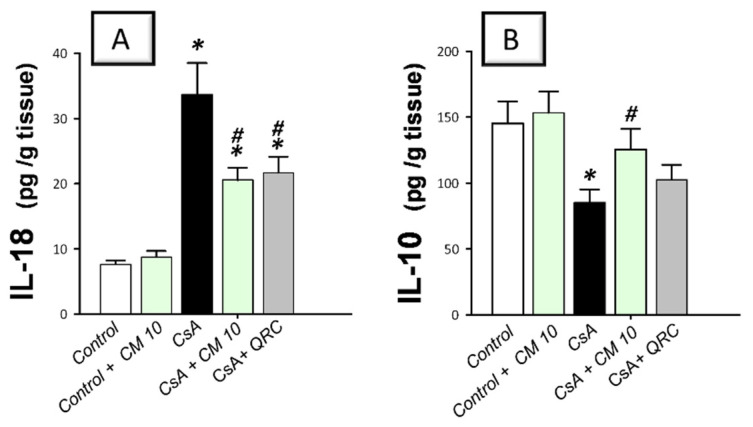
Effect of camel milk administration on the renal pro-inflammatory IL-18 and anti-inflammatory IL-10 cytokine levels in cyclosporine-evoked renal damage in rats. (**A**) Camel milk lowers the renal level of interleukin-18 (IL-18). (**B**) Camel milk augments the renal level of interleukin-10 (IL-10). Values are displayed as mean ± SEM, for *n* = 8 samples per each group (one sample from each rat). * Significance vs. control values at *p* < 0.05; *^#^* Significance vs. CsA values at *p* < 0.05. CsA, cyclosporine (20 mg/kg/day, s.c., for 3 weeks); CM 10, camel milk (10 mL/kg/day, by gavage, for 3 weeks); QRC; the reference antioxidant quercetin (50 mg/kg/day, by gavage, for 3 weeks).

To delineate the inflammatory responses in the kidney tissues that were instigated by CsA, the production of inflammatory cytokines was investigated. Relative to the control group, CsA administration provoked a remarkable renal inflammation, as indicated by a significant increase of renal pro-inflammatory signals, including MCP-1 (4.1-fold), TNF-α (3.5-fold), IL-1β (3.4-fold; Figure 1), and IL-18 (4.4-fold; Figure 2A). Meanwhile, CsA diminished the renal anti-inflammatory signals, such as IL-10 to a nearly two-fold decrease, as depicted in Figure 2B. Interestingly, camel milk elicited significant inhibition of the pro-inflammatory signals, as marked by the suppression of MCP-1 (61.4%), TNF-α (57.5%), IL-1β (54.7%), and IL-18 (39%), relative to the renal injury group. In favor of combating renal inflammation, camel milk also augmented the IL-10 levels to 86.5% of their control values. Similarly, quercetin reversed these changes and suppressed the inflammatory responses. Together, camel milk curbed the renal inflammation evoked by CsA by suppressing the pro-inflammatory cytokine generation and augmenting the anti-inflammatory IL-10, thereby, participating in the attenuation of CsA-provoked renal injury.

### 3.3. Camel Milk Inhibits the Renal NF-κB Activation Triggered by CsA in Rats

**Figure 3:**
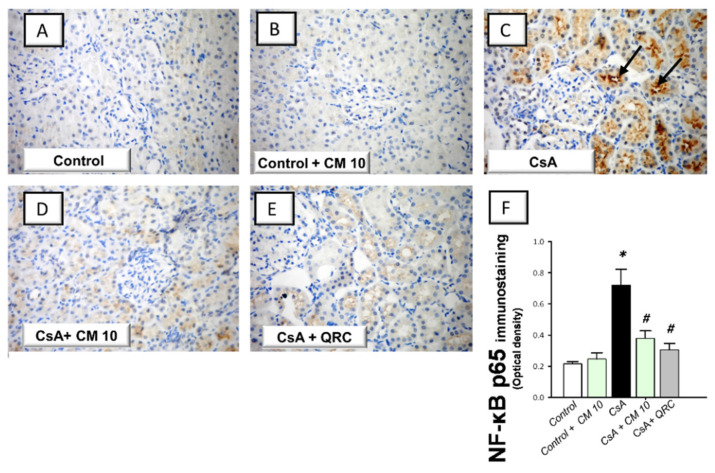
Effect of camel milk administration on the renal protein expression of activated nuclear factor kappa Bp65 (NF-κB p65) in cyclosporine-evoked renal damage in rats. (**A**–**E**) Evaluation of the renal expression of NF-κBp65 by immunohistochemical staining (×400 magnification). Representative photomicrographs depicting minimal expression of NF-κBp65 in the control (**A**) and camel milk-treated control (**B**). CsA administration triggered an increased expression of the activated NF-κBp65 protein subunit (positive brown staining is indicated by an arrow; (**C**), which was attenuated in camel milk-treated renal injury group (**D**) and quercetin-treated renal injury group (**E**). (**F**) Quantification of the immunostaining of NF-κBp65 (optical density) in all groups. Values are displayed as mean ± SEM, for 4 samples per group. The immunostaining was examined across 6 non-overlapping fields per section. * Significance vs. control values at *p* < 0.05; *^#^* Significance vs. CsA values at *p* < 0.05. CsA, cyclosporine (20 mg/kg/day, s.c., for 3 weeks); CM 10, camel milk (10 mL/kg/day, by gavage, for 3 weeks); QRC; the reference antioxidant quercetin (50 mg/kg/day, by gavage, for 3 weeks).

**Figure 4:**
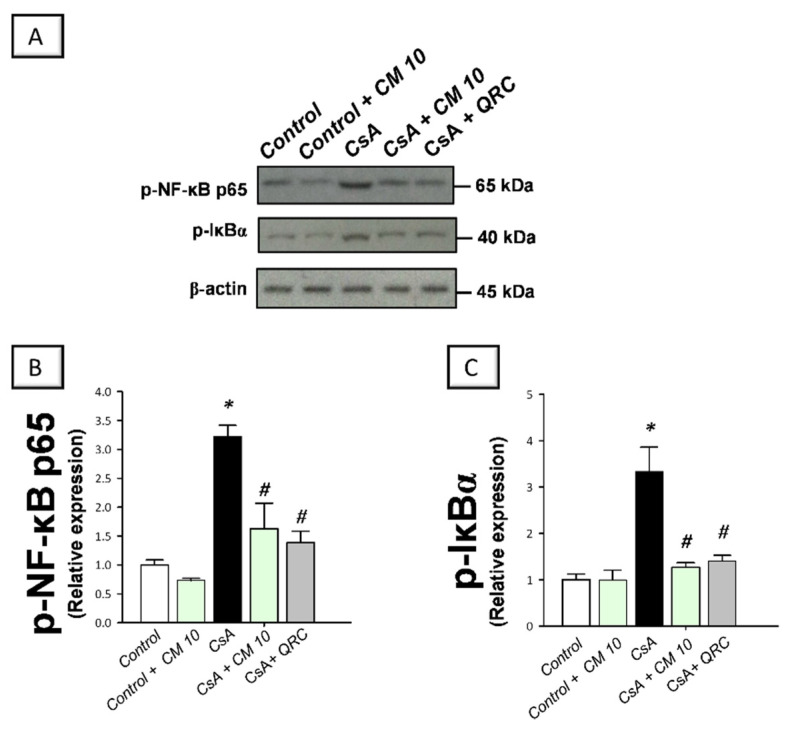
Effect of camel milk administration on the activation of the nuclear factor kappa B (NF-κB) pathway in cyclosporine-evoked renal damage in rats. (**A**) Representative immuno-blots that demonstrate the inhibition of the nuclear factor-kappa B pathway by camel milk, as evidenced by lowered phosphorylation of NF-κBp65 (Ser536; upper panel) and the inhibitory protein for NF-κB (IκBα; Ser32; lower panel). (**B**) Quantification of p-NF-κBp65 relative protein expression. (**C**) Quantification of p-IκBα relative protein expression. Values for the Western blotting are displayed as mean ± SEM, for *n* = 3 independent experiments per each group. The X-ray films were photographed with an HD-Nikon camera and the densitometric analysis of protein bands was carried out using Image J software. Equal loading/protein transfer was proven by probing with anti-β-actin. * Significance vs. control values at *p* < 0.05; *^#^* Significance vs. CsA values at *p* < 0.05. CsA, cyclosporine (20 mg/kg/day, s.c., for 3 weeks); CM 10, camel milk (10 mL/kg/day, by gavage, for 3 weeks); QRC; the reference antioxidant quercetin (50 mg/kg/day, by gavage, for 3 weeks). Original western blot images are shown in Figure S1.

The molecular mechanisms pertaining to CsA-triggered inflammatory events were explored by examining the pro-inflammatory NF-κB pathway, a crucial pro-inflammatory pathway in the pathogenesis of CsA nephrotoxicity [4,5,6]. Relative to the control group, CsA administration instigated a significant activation of the NF-κB pathway, as proven with an upregulated NF-κB p65 protein expression (3.35-fold) that was detected by immunohistochemistry (Figure 3). Meanwhile, the immunoblotting demonstrated a significant increase of p-NF-κB p65 (Ser356; 3.22-fold) and p-IκBα (3.33-fold) protein expression, relative to the control group (Figure 4). Interestingly, camel milk downregulated the protein expression activated NF-κB p65 (by 47.4%; Figure 3) and the phosphorylation of NF-κB p65 (by 49.5%) and IκBα (by 61.9%) proteins, relative to the renal injury group (Figure 4). These data point to the notion that camel milk’s suppression of the NF-κB pathway contributes to the mitigation of renal inflammation and damage. 

### 3.4. Camel Milk Inhibits the MAPK Transduction in CsA-Triggered Renal Injury in Rats

**Figure 5:**
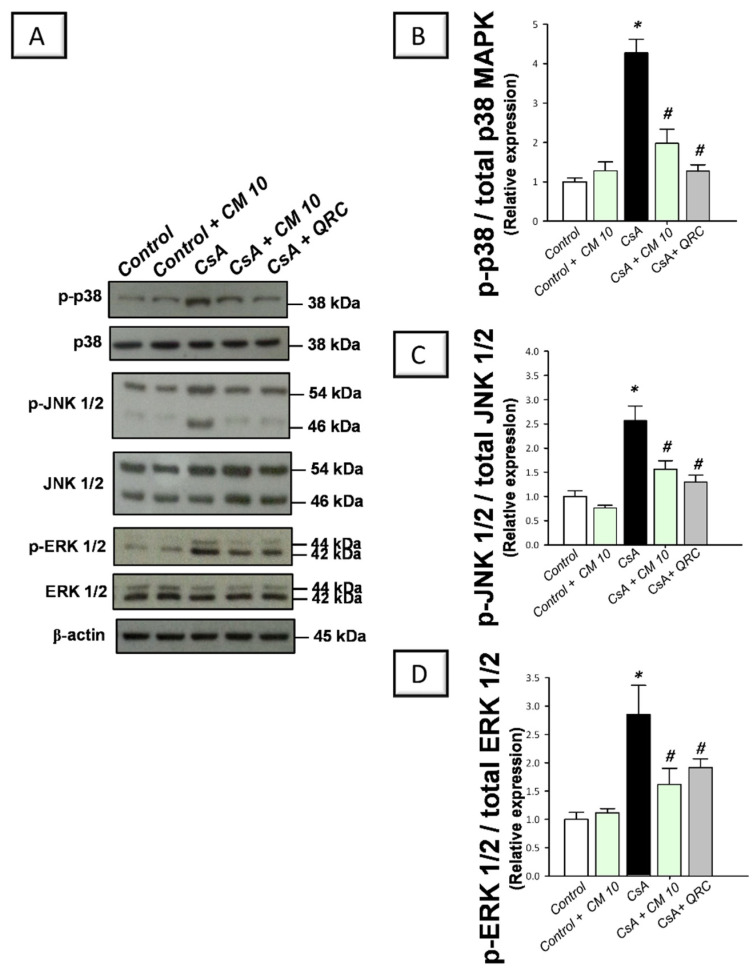
Effect of camel milk administration on the transduction of mitogen-activated protein kinase (MAPK) pathway in cyclosporine-evoked renal damage in rats. (**A**) Representative immuno-blots that demonstrate the suppression of the mitogen-activated protein kinase (MAPK) pathway by camel milk, as evidenced by lowered expression of phospho-p38 (p-p38) MAPK (Thr180/Tyr182)/total p38 MAPK, phospho-c-Jun N-terminal kinase 1/2 (p-JNK1/2 (Thr 183/185)/total JNK1/2, and phospho- extracellular signal-regulated kinase 1/2 (p-ERK1/2 (Thr202/Tyr204)/total ERK1/2 ratios. (**B**) Band intensity quantification of p38MAPK (Thr180/Tyr182)/total p38 MAPK relative protein expression. (**C**) Band intensity quantification of p-JNK1/2 (Thr 183/185)/total JNK1/2 relative protein expression. (**D**) Band intensity quantification of p-ERK1/2 (Thr202/Tyr204)/total ERK1/2 relative protein expression. Values for the Western blotting are displayed as mean ± SEM, for *n* = 3 independent experiments per each group. The X-ray films were photographed with an HD-Nikon camera and the densitometric analysis of protein bands was carried out using Image J software. Equal loading/protein transfer was proven by probing with anti-β-actin. * Significance vs. control values at *p* < 0.05; *^#^* Significance vs. CsA values at *p* < 0.05. CsA, cyclosporine (20 mg/kg/day, s.c., for 3 weeks); CM 10, camel milk (10 mL/kg/day, by gavage, for 3 weeks); QRC; the reference antioxidant quercetin (50 mg/kg/day, by gavage, for 3 weeks). Original western blot images are shown in Figure S2.

The molecular events of renal inflammation were further dissected by examining the upstream pro-inflammatory MAPK cascade which is essential for mediating the CsA-induced renal damage [3,7,8]. Relative to the control group, CsA administration instigated a significant activation of the MAPK pathway, as evidenced by increased phosphorylation of its three subfamily member proteins, including, p-p38MAPK/total p38 MAPK (4.27-fold), p-JNK1/2/total JNK1/2 (2.56-fold), and p-ERK1/2/total ERK1/2 (2.85-fold) ratios, relative to the control group (Figure 5). Parallel to the NF-κB pathway inhibition, camel milk also counteracted the activation of renal MAPK signaling, as seen by downregulated expression of p-p38MAPK/total p38 MAPK (by 53.9%), p-JNK1/2/total JNK1/2 (by 39.1%), and p-ERK1/2/total ERK1/2 (43.3%), relative to the renal injury group. Together, these findings demonstrate that the inhibition of MAPK transduction is involved in the ameliorating effects of camel milk against CsA-evoked renal injury. 

### 3.5. Camel Milk Downregulates the MMP-2 and MMP-9 Protein Expression in CsA-Triggered Renal Injury in Rats

**Figure 6:**
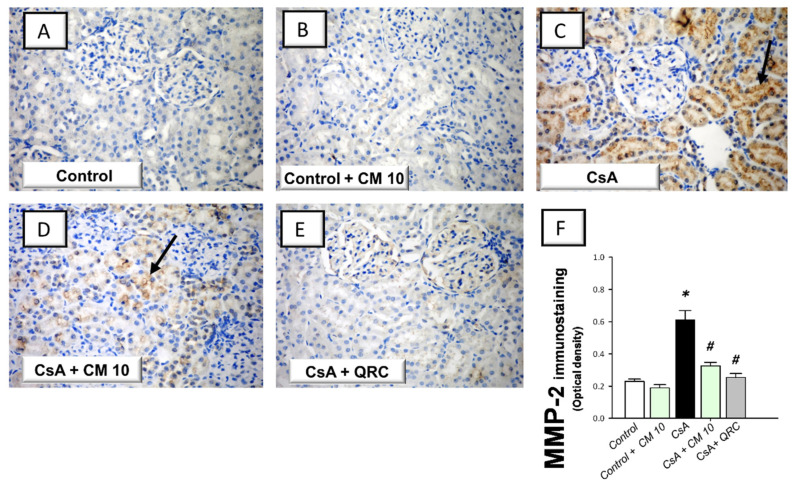
Effect of camel milk administration on the renal protein expression of MMP-2 in cyclosporine-evoked renal damage in rats. (**A**–**E**) Evaluation of the renal expression of matrix metalloproteinase-2 (MMP-2) by the immunohistochemical staining (×400 magnification). Representative photomicrographs depicting minimal expression of MMP-2 in the control (**A**) and camel milk-treated control (**B**) groups. CsA administration triggered an increased expression of MMP-2 (positive brown staining is indicated by an arrow; (**C**), which was attenuated in camel milk-treated renal injury group (**D**) and quercetin-treated renal injury group (**E**). (**F**) Quantification of the immunostaining of MMP-2 (optical density) in all groups. Values are displayed as mean ± SEM, for 4 samples per group. The immunostaining was examined across 6 non-overlapping fields per section. * Significance vs. control values at *p* < 0.05; *^#^* Significance vs. CsA values at *p* < 0.05. CsA, cyclosporine (20 mg/kg/day, s.c., for 3 weeks); CM 10, camel milk (10 mL/kg/day, by gavage, for 3 weeks); QRC; the reference antioxidant quercetin (50 mg/kg/day, by gavage, for 3 weeks).

**Figure 7:**
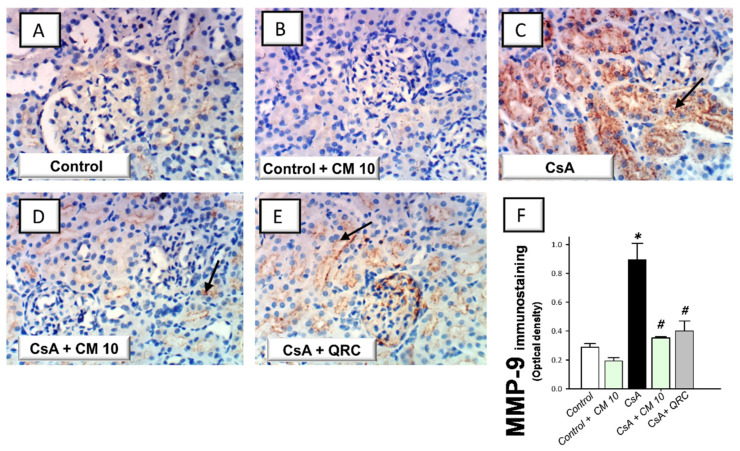
Effect of camel milk administration on the renal MMP-9 protein expression in cyclosporine-evoked renal damage in rats. (**A**–**E**) Evaluation of the renal expression of matrix metalloproteinase-9 (MMP-9) by the immunohistochemical staining (×400 magnification). Representative photomicrographs depicting minimal expression of MMP-9 in the control (**A**) and camel milk-treated control (**B**) groups. CsA administration triggered an increased expression of MMP-9 (positive brown staining is indicated by an arrow; (**C**), which was attenuated in camel milk-treated renal injury group (**D**) and quercetin-treated renal injury group (**E**). (**F**) Quantification of the immunostaining of MMP-9 (optical density) in all groups. Values are displayed as mean ± SEM, for 4 samples per group. The immunostaining was examined across 6 non-overlapping fields per section. * Significance vs. control values at *p* < 0.05; *^#^* Significance vs. CsA values at *p* < 0.05. CsA, cyclosporine (20 mg/kg/day, s.c., for 3 weeks); CM 10, camel milk (10 mL/kg/day, by gavage, for 3 weeks); QRC; the reference antioxidant quercetin (50 mg/kg/day, by gavage, for 3 weeks).

The extracellular matrix degradation events associated with renal inflammation were investigated by the immunohistochemical staining of the matrix metalloproteinase-2 (MMP-2) and matrix metalloproteinase-9 (MMP-9). CsA evoked an enhanced expression of MMP-2 (2.66-fold) and MMP-9 (3.11-fold), relative to the control group (Figure 6 and Figure 7, respectively). Interestingly, camel milk attenuated the expression of these degradative signals, as seen by 46.6% and 60.6% inhibition of the two matrix metalloproteinases, respectively, relative to the renal injury group. Together, the observations reveal that the downregulation of the extracellular matrix degradative signals is intimately linked to camel milk’s attenuation of CsA renal injury. 

### 3.6. Camel Milk Reverses the Oxidative Insult Triggered by CsA in Rats

**Figure 8:**
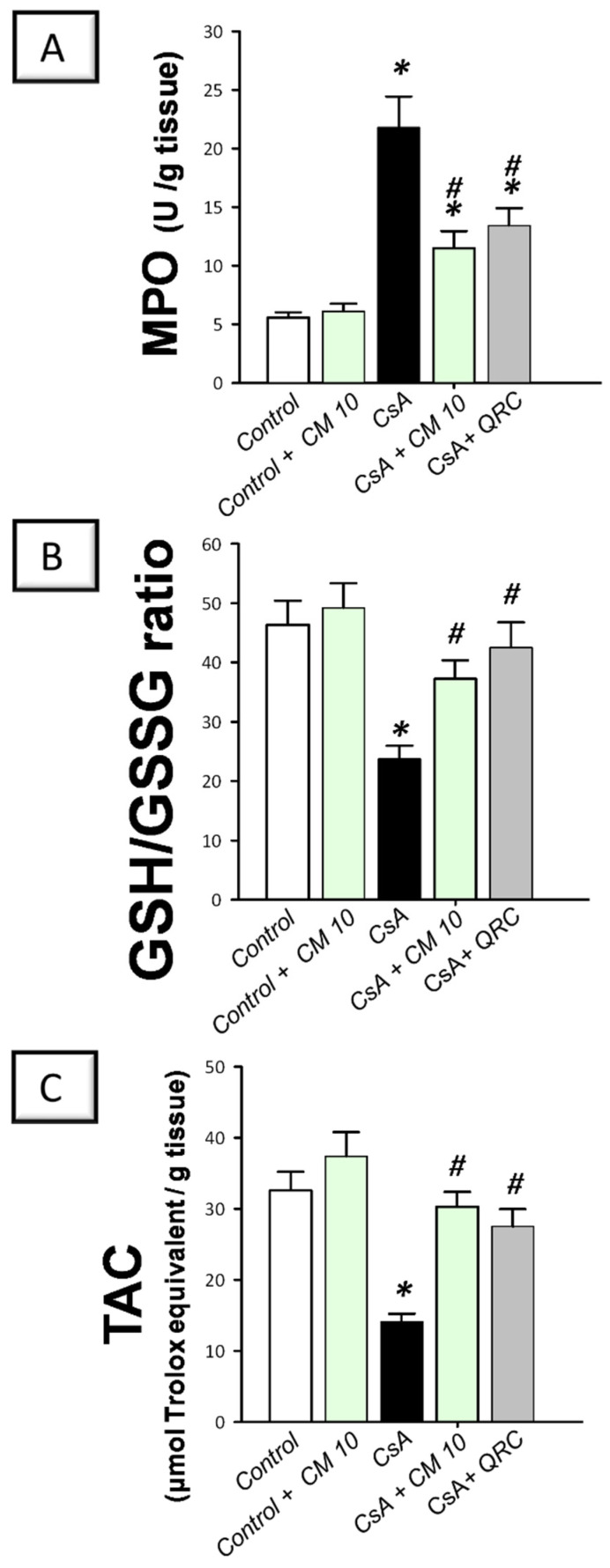
Effect of camel milk administration on the renal oxidative aberrations in cyclosporine-evoked renal damage in rats. (**A**) Camel milk lowers the renal activity of myeloperoxidase (MPO). (**B**) Camel milk boosts the renal reduced/oxidized glutathione (GSH/GSSG) ratio. (**C**) Camel milk augments the renal level of the total antioxidant capacity (TAC). Values are displayed as mean ± SEM, for *n* = 8 samples per each group (one sample from each rat). * Significance vs. control values at *p* < 0.05; *^#^* Significance vs. CsA values at *p* < 0.05. CsA, cyclosporine (20 mg/kg/day, s.c., for 3 weeks); CM 10, camel milk (10 mL/kg/day, by gavage, for 3 weeks); QRC; the reference antioxidant quercetin (50 mg/kg/day, by gavage, for 3 weeks).

To explore the oxidative milieu of the kidney tissues, the renal myeloperoxidase (MPO) activity, reduced/oxidized glutathione (GSH/GSSG) ratio, and the content of the total antioxidant capacity (TAC) were investigated. Relative to the control group, CsA administration resulted in a significant increase of the renal MPO activity (3.9-fold; Figure 8); which signifies a marked neutrophil infiltration to the kidney tissue, since MPO has been considered as a reliable marker for the invasion of tissues by polymorphonuclear leukocytes [30]. Meanwhile, cyclosporine lowered the GSH/GSSG ratio and TAC to reach 51.2% and 43.2%, respectively, relative to the control group. These aberrations were reversed by camel milk which suppressed the MPO activity by 47.2% and augmented the GSH/GSSG ratio and TAC to reach 80.3% and 93%, respectively, relative to the control values. Together, these observations reveal that the augmentation of renal antioxidant capacity and reversal of oxidative insult by camel milk mediate, at least in part, the ability to attenuate CsA-evoked renal damage.

## 4. Discussion

**Figure 9:**
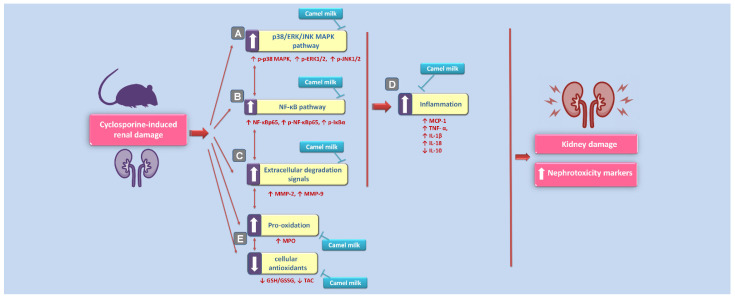
An overview of the suggested molecular mechanisms that interceded the beneficial impact of camel milk in cyclosporine-evoked renal injury. According to the current findings, the proposed mechanisms pertaining to the attenuation of cyclosporine-evoked nephrotoxicity are: (**A**) Camel milk inhibited the upstream pro-inflammatory p38/ERK/JNK MAPK pathway, as revealed by lowered phosphorylation of the three subfamilies of MAPK (p38 MAPK, ERK1/2, and JNK1/2). (**B**) Meanwhile, camel milk inhibited the activation of the pro-inflammatory NF-κB pathway, as seen by downregulated expression of activated NF-κBp65 alongside p-NF-κBp65 and p-IκBα proteins. (**C**) Camel milk lowered the renal expression of the extracellular degradation signals matrix metalloproteinases (MMP-2 and MMP-9). (**D**) Camel milk inhibited renal inflammation, as evidenced by lowered renal levels of MCP-1, TNF-α, IL-1β, and IL-18 pro-inflammatory cytokines and enhanced the production of the anti-inflammatory signal IL-10. (**E**) The curtailing of renal oxidative stress and augmentation of the antioxidant capacity also contributed to the amelioration of cyclosporine-evoked renal damage. The blunt arrows indicate inhibition, and the solid arrows indicate activation.

Several interventions have been introduced to halt the pathogenesis of cyclosporine-evoked renal failure; a serious complication during the therapy with cyclosporine in patients with autoimmune diseases and organ transplantation [1,2,3,4]. The present work suggests that camel milk is a promising natural agent for counteracting cyclosporine-triggered nephrotoxicity. In perspective, the cellular and molecular mechanisms of camel milk were elucidated where camel milk curbed the renal inflammation through the inhibition of the upstream p38/ERK/JNK MAPK pathway and its downstream NF-κB cascade and associated production of pro-inflammatory cytokines and matrix metalloproteinases (Figure 9). Notably, camel milk significantly mitigated the renal damage at the dose of 10 mL/kg in rats. The human equivalent dose (HED) [31] for this dose is 1.63 mL/kg in humans (about 114 mL of camel milk for a 70-kg adult human), which is well-tolerated by humans. Worthy to mention that the contribution of camel milk’s lactoferrin and other antioxidant micronutrients to the mitigation of cyclosporine-induced renal damage seems significant [18,32,33]. 

The current set of experiments revealed that cyclosporine induced marked nephrotoxicity, as seen by significant increase of serum creatinine and BUN, and renal protein expression of KIM-1. These findings are in agreement with ample evidence from literature [1,2]. Notably, the upregulated renal protein expression of KIM-1 suggests a significant renal tubular injury and has been envisioned as a compensatory response in the renal tissues to guard against tubular cell apoptosis and to enhance the re-epithelization of the kidney tubules [29]. Virtually, KIM-1, a transmembrane protein with immunoglobulin and mucin domains, is a well-recognized early marker of renal tubular damage which can also predict the progression of acute kidney injury [14,29]. Herein, we report that camel milk significantly lowered these nephrotoxicity markers, confirming its reno-protective impact against cyclosporine-evoked renal injury. Multiple reports have documented the reno-protective ability of camel milk in the clinical setting against microalbuminuria in diabetes mellitus nephropathy and in vivo rodent models of toxicant-induced renal injury [13,14] and diabetic nephropathy [34]. 

The present findings reveal an exaggerated renal inflammatory response triggered by the cyclosporine insult in rats. This was manifested by significant increase of the renal MCP-1, TNF-α, IL-1β, and IL-18 pro-inflammatory cytokines, alongside the MPO enzyme. The observed spike in MPO activity indicates renal invasion with inflammatory leukocytes and parenchymal structural derangements [3,35]. Meanwhile, the influx of activated macrophages/monocytes generates superfluous amounts of several pro-inflammatory cytokines, such as MCP-1, TNF-α, IL-1β, and IL-18; events that initiate and perpetuate exaggerated inflammatory responses in several renal injury models [6,21,35,36]. IL-18 has been associated with enhanced expression of the pro-inflammatory NF-κB p65 and adhesion molecule ICAM-1 [37]. The current data also demonstrated a decline of renal IL-10, an anti-inflammatory cytokine released by monocytes and lymphocytes to antagonize the deleterious effects of proinflammatory cytokine overshooting [3,6,35].

Current data demonstrated that camel milk displayed marked anti-inflammatory and immunomodulatory actions, as proven with the suppression of MCP-1, TNF-α, IL-1β, and IL-18 pro-inflammatory cytokine overshooting with the enhancement of IL-10 in the renal tissues. These data are in concert with the previously reported anti-inflammatory actions of camel milk in rodent models of rheumatoid arthritis [10], inflammatory bowel disease [38], steatohepatitis [11], alcohol-induced hepatic injury [39], and wound healing [12]. A plausible mechanism that may explain camel milk’s suppression of the leukocyte infiltration marker MPO is the observed decline of TNF-α levels which is recognized for triggering leukocyte recruitment via increasing the expression of ICAM and P-selectin adhesion molecules [3,40]. Additionally, lactoferrin, a unique component of camel milk, has demonstrated a marked efficacy for halting the production of the proinflammatory cytokines and chemokines in mononuclear cells [3,32].

Ample evidence has revealed that the MAPK pathway is a crucial signaling cascade in the pathogenesis of cyclosporine-triggered renal injury [7], culminating in glomerular dysfunction and marked nephrotoxicity [3]. In fact, ample evidence has revealed that the activation of the three subfamilies of MAPKs, namely, p38 MAPK, ERK1/2, and JNK1/2, is involved in mediating cyclosporine-evoked renal damage [3,8]. In this regard, a study by Martin-Martin et al. [7] has revealed that the activation of ERK 1/2 is a central event in cyclosporine-induced increased paracellular permeability in vitro, as demonstrated in Madin-Darby canine kidney (MDCK) cells and LLC-PK1 porcine proximal tubular epithelial cells. In perspective, these deleterious effects were mediated by ERK 1/2 activation and associated disturbance of the tight-junction claudin-1 protein expression [3]. Additionally, the human mesangial cell model has also demonstrated that cyclosporine instigates excessive ROS production which activates the ERK1/2 pathway by triggering its phosphorylation [41]. Yet, the exact in vivo role of MAPK pathway in the pathogenesis of cyclosporine-induced renal damage in rodent models has not adequately characterized. Herein, the present work provides in vivo evidence for the activation of the three subfamilies of MAPKs pathway in the kidneys of Wistar albino rats in response to cyclosporine insult. Notably, the activation of p38 MAPK and JNK1/2 pathways has been reported to instigate the generation of pro-inflammatory cytokines and exaggerated inflammatory responses in several rodent models of kidney injury [7,8]. The crosstalk between MAPKs pathway and its downstream effector NF-κB has been demonstrated where ERK 1/2 phosphorylation can activate the NF-κB by releasing the inhibitory IκBα subunit with consequent liberation of the active NF-κB p65 subunit that serves as a transcription factor for the production of an array of pro-inflammatory signals, including the inflammatory cytokines [3,6].

The current data also demonstrated that the NF-κB pathway activation is implicated in meditating cyclosporine-induced renal toxicity. Previous studies have reported the in vitro and in vivo activation of the NF-κB signaling in response to cyclosporine insult [5,42,43]. Several stress signals have been characterized to trigger the activation of the NF-κB pathway, including excessive ROS, pro-inflammatory cytokines, and activated ERK1/2 MAPK pathway [7,8,21]. The crosstalk between NF-κB and p38 MAPK has been previously characterized in acute kidney injury models [3,44]. Notably, the activation of the NF-κB pathway liberates the NF-κB p65 that binds to the promoter region of several pro-inflammatory signal genes, resulting in excessive production of inflammatory mediators. Thus, the detection of activated NF-κB p65 subunit serves as an index for the activation of the NF-κB pathway [3,21].

Regarding the extracellular matrix metalloproteinases, the current study demonstrated an upregulated expression of MMP-2 and MMP-9 in cyclosporine-evoked renal damage in rats. Evolving evidence exists that oxidative stress signals trigger increased expression and activation of MMP-2 and MMP-9 signals that have been demonstrated in several renal pathologies in rodents [45]. In this regard, the two metalloproteinases activate the inflammatory cells, culminating in chemotaxis and associated production of the pro-inflammatory cytokines. In perspective, MMP-9 has been linked to triggering the inflammatory response and excessive neutrophil chemotaxis through its ability to generate extracellular collagen fragments in the kidney tissue [45,46]. Notably, the co-expression of MMP-9 with the neutrophil gelatinase-associated lipocalin in neutrophils provokes the prolongation of its action and exacerbates the renal pathology [46]. Generally, agents that can elicit effective suppression of the MMP activities have been characterized as beneficial strategies for alleviating renal injury in the majority of renal pathologic models [45].

Interestingly, camel milk demonstrated multi-pronged anti-inflammatory effects against cyclosporine-evoked renal injury, as proven with significant suppression of the upstream p38 MAPK, JNK1/2, and ERK1/2 pro-inflammatory components of the MAPK pathway. In addition, camel milk also curbed the NF-κB pathway activation, as seen by lowered phosphorylation of the NF-κB p65 and IκBα together with downregulated expression of the activated NF-κB p65 subunit. In fact, the observed multipronged suppression of these pro-inflammatory signals affirms the marked anti-inflammatory features of camel milk which is in agreement with previous studies [10,12,14]. The inhibition of MAPK cascade together with the NF-κB pathway has been envisioned as a reliable anti-inflammatory strategy for counteracting the renal damage in multiple renal injury models in vivo [21,35]. In perspective, camel milk has shown remarkable efficacy for inhibiting the MAPK pathway in rodent models of 5-fluorouracil-induced renal injury [14], rheumatoid arthritis [10], and LPS-triggered respiratory distress [47]. Notably, the blockade of p38 MAPK and JNK1/2 has demonstrated notable attenuation of the renal tubular cell death and renal failure in diverse kidney injury pathologies [3,8,48,49]. In the same context, camel milk has been reported to halt the activation of NF-κB pathway and improve wound healing in rats with diabetes mellitus [12].

A plethora of reports have revealed that the exaggerated oxidative stress is a central element in the pathogenesis of cyclosporine-induced renal damage [3,50]. Overshooting of ROS has been reported in human renal mesangial cells in vitro [3] and rodent models of nephrotoxicity, with excessive production of hydrogen peroxide, superoxide anions, hydroxyl radicals, and peroxynitrite [3,4]. Notably, cyclosporine has also been reported to deplete the renal cellular antioxidants, as evidenced herein and in former studies [1,2,4]. Additionally, the observed elevation of MPO activity contributes to cyclosporine-evoked renal oxidative injury through the excessive production of the cytotoxic oxidant hypochlorous acid [30]. Interestingly, the present work revealed that camel milk curtailed the MPO activity and augmented the GSH/GSSG ratio and TAC, culminating in the attenuation of cyclosporine-evoked renal damage. These observations are in agreement with previous studies that confirmed the salient anti-oxidant activity of camel milk for the mitigation of several toxicant-induced renal damage [13,14]. The observed inactivation of MAPK pathway and NF-κB cascade in the current study are, at least partly, implicated in the mitigation of renal oxidative stress, since the two pathways have been reported to drive excessive ROS production [14,35,36]. Meanwhile, lactoferrin, a unique antioxidant component of camel milk, plays a central role in counteracting renal oxidative stress by curtailing hydroxyl radical production and scavenging of free iron in injured tissues [33,51].

## 5. Conclusions

The present work suggests that camel milk serves as a promising intervention against cyclosporine-evoked renal insult. These beneficial impacts were chiefly interceded by the multi-pronged suppression of renal inflammation through the inactivation of the three subfamilies of MAPKs (p38 MAPK, ERK1/2, and JNK1/2), NF-κB cascade, and associated pro-inflammatory cytokine and matrix metalloproteinases production. Supplemental experiments are needed to provide additional insights into the precise cellular and molecular mechanisms that mediated camel milk ameliorative effects using multiple doses of camel milk. Additionally, exploration of the potential of lactoferrin, a bioactive component of camel milk with potent anti-inflammatory/antioxidant features, to attenuate cyclosporine-evoked renal damage is warranted. 

## Figures and Tables

**Table 1:** Effect of the administration of camel milk on body weight change and renal function biomarkers in cyclosporine-evoked renal damage in rats.

	Control	Control + CM 10	CsA	CsA + CM 10	CsA + QRC
Change of body weight (g) (final weight − initial weight)	16.12 ± 2.08	20.87 ± 3.45	−14.38 ± 1.79 *	5.32 ± 1.09 *^,#^	8.96 ± 1.52 ^#^
Serum creatinine (mg/dL)	0.38 ± 0.04	0.34 ± 0.04	1.05 ± 0.13 *	0.61 ± 0.05 ^#^	0.72 ± 0.09 ^#^
Serum BUN (mg/dL)	24.69 ± 1.34	30.86 ± 2.76	64.86 ± 5.59 *	37.98 ± 3.40 ^#^	45.24 ± 4.18 ^#^
Renal KIM-1 (pg/g tissue)	12.87 ± 1.07	11.65 ± 1.259	33.65 ± 3.88 *	17.28 ± 2.16 ^#^	22.78 ± 1.56 ^#^

Camel milk inhibits the loss of body weight and improves the renal dysfunction biomarkers triggered by CsA, including serum creatinine and blood urea nitrogen (BUN), alongside the renal protein expression of the kidney injury molecule-1 (KIM-1). Cyclosporine (20 mg/kg/day) was subcutaneously injected for 3 consecutive weeks and camel milk (10 mL/kg/day) was orally administered by gavage for the same period. Values are displayed as mean ± SEM, for *n* = 8 samples per each group (one sample from each rat). * Significance vs. control values at *p* < 0.05; ^#^ Significance vs. CsA values at *p* < 0.05. CsA, cyclosporine (20 mg/kg/day, s.c., for 3 weeks); CM 10, camel milk (10 mL/kg/day, by gavage, for 3 weeks); QRC; the reference antioxidant quercetin (50 mg/kg/day, by gavage, for 3 weeks).

## Data Availability

Data available upon request.

## Supplementary Materials

The following are available online at https://www.mdpi.com/article/10.3390/biology10050442/s1, Figure S1: The X-ray film images for Western blot represent the expression level of p-NF-κBp65, p-IκBα, and the loading control β actin described in Figure 4, Figure S2: The X-ray film images for Western blot represent the expression level of p-p38 MAPK, total p38 MAPK, p-JNK1/2, total JNK1/2, p-ERK1/2, total ERK1/2, and the loading control β actin described in Figure 5.
