# Supplementary material for: Camel Milk Mitigates Cyclosporine-Induced Renal Damage in Rats: Targeting p38/ERK/JNK MAPKs, NF-κB, and Matrix Metalloproteinases
Source: Biology (Basel). 2021 May 17;10(5):442. doi: 10.3390/biology10050442 (PMC8156933; doi:10.3390/biology10050442)
Supplement: Supplementary file 1 [file biology-10-00442-s001.zip › biology-1176903-supplementary.pdf]

biology

MDPI

Supplementary material

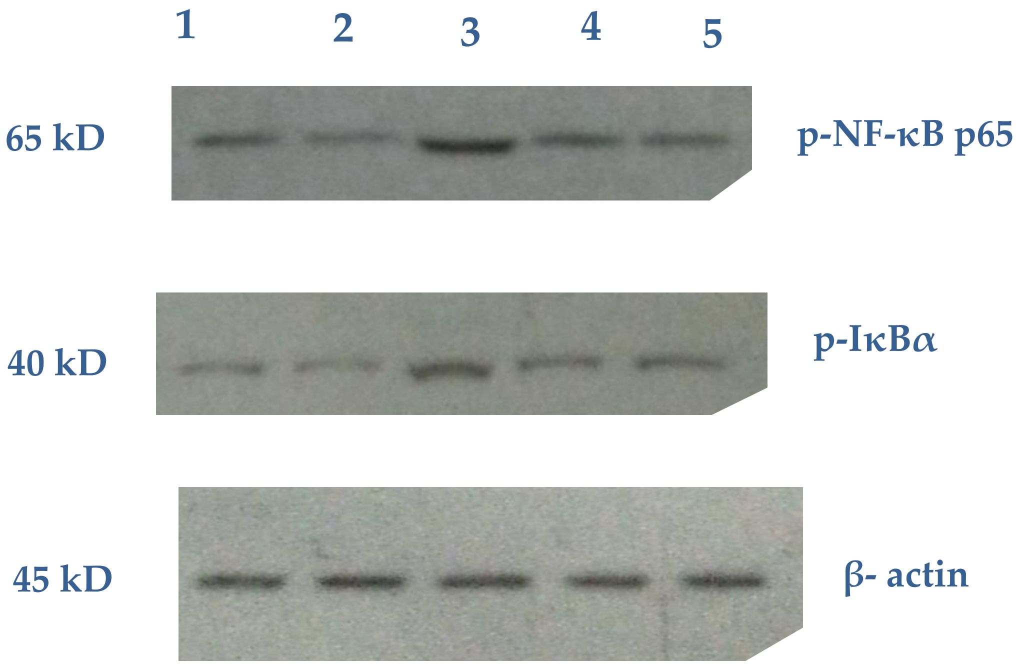

[1]: Control

[2]: Control + camel milk

[3]: Cyclosporine

[4]: Cyclosporine + Camel milk

[5]: Cyclosporine + Quercetin

Figure S1: The X-ray film images for Western blot represent the expression level of p-NF-κBp65 , p- IκBα, and the loading control β-actin (**Figure 4)**

biology MDPI

Supplementary material

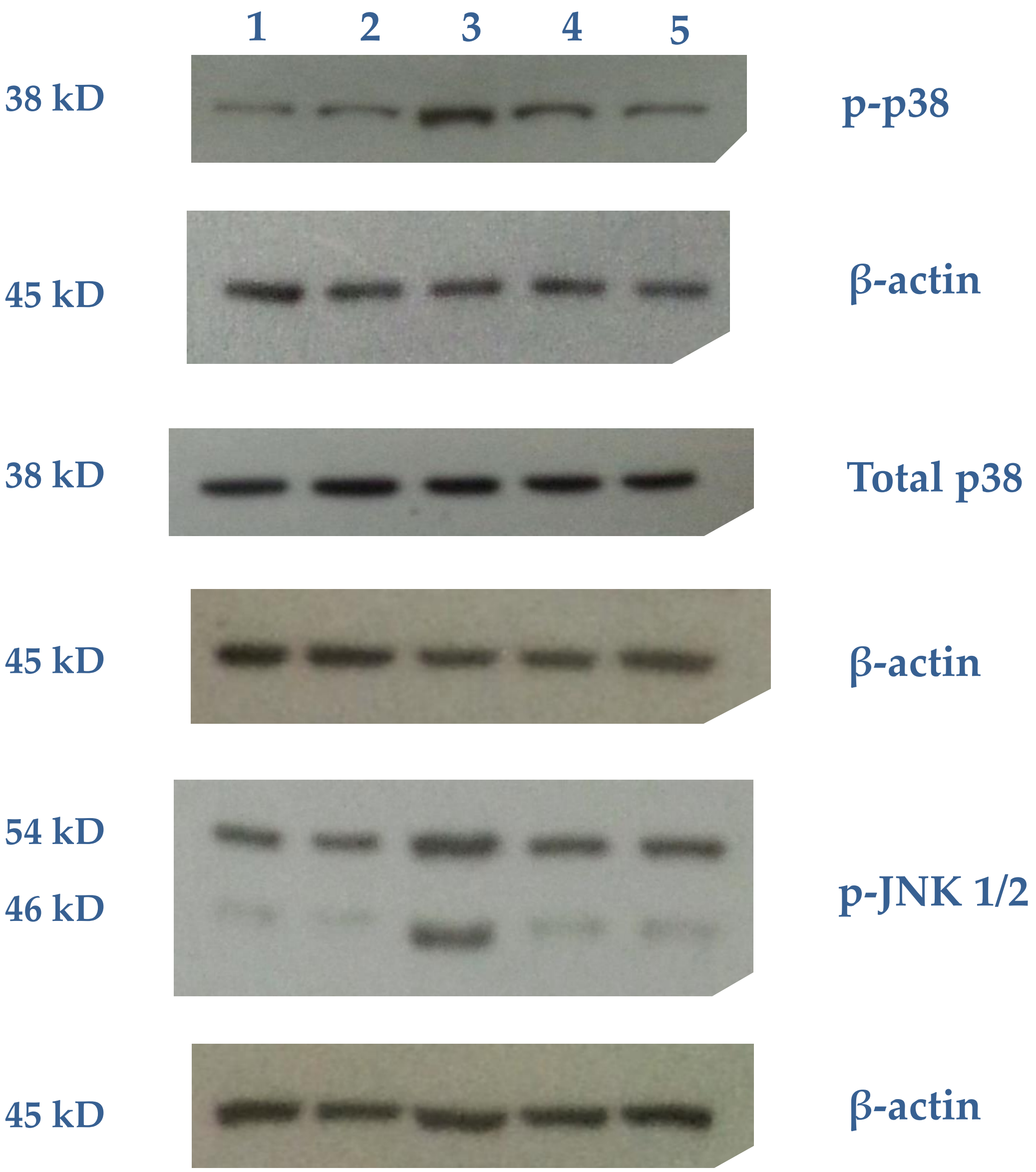

[1]: Control

[2]: Control + camel milk

[3]: Cyclosporine

[4]: Cyclosporine + Camel milk

[5]: Cyclosporine + Quercetin

Figure S2- A

Supplementary material

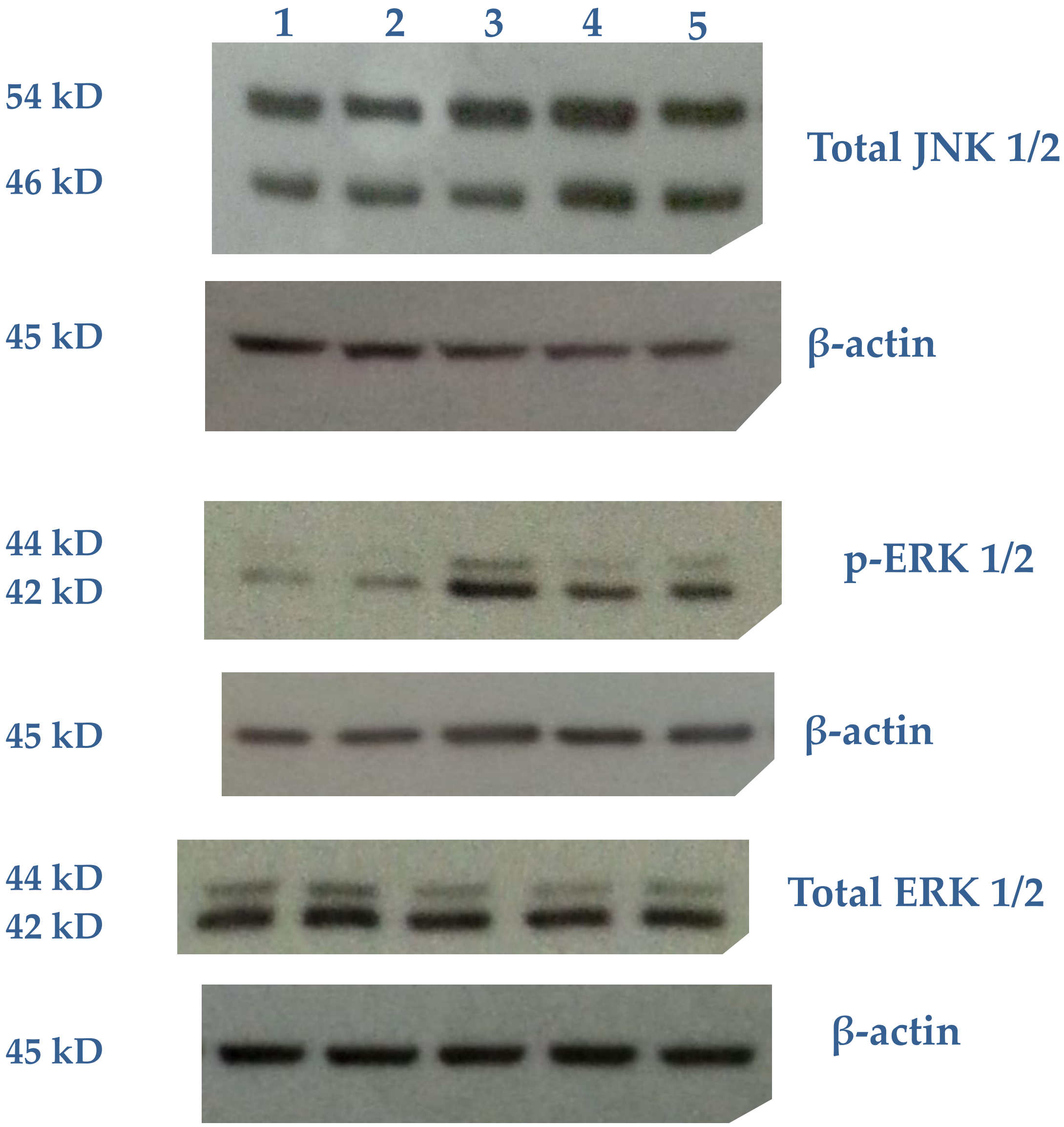

[1]: Control

[2]: Control + camel milk

[3]: Cyclosporine

[4]: Cyclosporine + Camel milk

[5]: Cyclosporine + Quercetin

Figure S2- B

Figure S2: The X-ray film images for Western blot represent the expression level of p-p38 MAPK, total p38 MAPK, p-JNK1/2, total JNK1/2, p-ERK1/2, total ERK1/2, and the loading control β-actin (**Figure 5).**
